# Validity of International Health Regulations in Reporting Emerging Infectious Diseases

**DOI:** 10.3201/eid1807.111608

**Published:** 2012-07

**Authors:** Michael Edelstein, David L. Heymann, Johan Giesecke, Julius Weinberg

**Affiliations:** London School of Hygiene and Tropical Medicine, London, UK (M. Edelstein, D.L. Heymann);; Chatham House, London (D.L. Heymann);; European Centre for Disease Control, Stockholm, Sweden (J. Giesecke); and; Kingston University, London (J. Weinberg)

**Keywords:** international health regulations, decision instrument, communicable diseases, emerging infectious diseases, reporting, notification, World Health Organization, sensitivity, specificity, diagnosis, reproducibility of results

## Abstract

Understanding which emerging infectious diseases are of international public health concern is vital. The International Health Regulations include a decision instrument to help countries determine which public health events are of international concern and require reporting to the World Health Organization (WHO) on the basis of seriousness, unusualness, international spread and trade, or need for travel restrictions. This study examined the validity of the International Health Regulations decision instrument in reporting emerging infectious disease to WHO by calculating its sensitivity, specificity, and positive predictive value. It found a sensitivity of 95.6%, a specificity of 38%, and a positive predictive value of 35.5%. These findings are acceptable if the notification volume to WHO remains low. Validity could be improved by setting more prescriptive criteria of seriousness and unusualness and training persons responsible for notification. However, the criteria should be balanced with the need for the instrument to adapt to future unknown threats.

## Abstract 2

Use of more prescriptive criteria and training of persons responsible for reporting could improve results.

The great influenza pandemic of 1918 and the increase in HIV/AIDS are 2 striking examples of the devastation and profound effect on human societies caused by emerging infectious diseases (EIDs) (*1*). The Institute of Medicine defines EIDs as “new, re-emerging, or drug-resistant infections whose incidence in humans has increased or whose incidence threatens to increase in the near future” (*2*). EIDs are a global phenomenon, with hotspots from which EIDs are more likely to appear, concentrated in low-latitude developing countries (*3*). EIDs are probably underreported, particularly in areas which have hotspots and also weak surveillance systems (*4*). A study in 2008 by Jones et al. reported 335 EIDs during 1940–2004 (*3*).

The purpose of the 2005 International Health Regulations (IHR) is to “help the international community prevent and respond to acute public health risks that have the potential to cross borders and threaten people worldwide” (*5*). This purpose includes development of an international reporting system, under which member states have a duty to report to the World Health Organization (WHO) “all events which may constitute a public health emergency of international concern” (*5*). These events are not limited to communicable diseases and can include contaminated food, chemical contamination of products or the environment, release of radionuclear material, or other toxic release (*6*). Events are reported to WHO by designated national focal points (NFPs) in each member state. WHO has designed a decision instrument contained in Annex 2 of the 2005 IHR (*7*) to assist with the notification process on the basis of an algorithm comprising 4 main criteria: the event has a serious public health effect, the event is unusual or unexpected, there is a major risk for international spread, and there is a major risk for international travel or trade restrictions. At least 2 of the criteria must be satisfied for an event to be notifiable.

An IHR expert committee suggested regular evaluations of the notification process (*8*). However, the only published evaluation of the Annex 2 decision instrument is a reliability study that analyzed NFPs notification concordance (*9*). This study also reported a sensitivity of 80% (on the basis of 5 events) and a specificity of 50% (on the basis of 4 events). Although the study reported a high reliability, the number of events was too low to adequately assess the sensitivity and specificity of the decision instrument. A 2008 WHO technical report on Annex 2 (*10*) mentions a 2006 workshop assessing the decision instrument validity and finding a sensitivity of 100% and a specificity of 55% on the basis of 10 events. There were no details on the methods used and the study results were not published.

The aim of this study was to evaluate the predictive validity of the Annex 2 decision instrument. We focused on EIDs by applying screening test evaluation methods to the IHR Annex 2 decision instrument and estimated its sensitivity, specificity, and positive predictive value (PPV).

## Methods

The sensitivity, specificity, and PPV of the Annex 2 decision instrument were calculated by asking an investigator to decide whether each event in a series of historical EID events would have been reported to WHO by using the criteria of the instrument. A panel of 3 internationally recognized EID and IHR experts, who were independent of the notifying investigator, was then asked whether each event was truly of international public health concern. The sensitivity, specificity, and PPV of the decision instrument were then calculated by cross-tabulating the outcome of the notification process and the true outcome of each event (taken as the expert panel consensus decision) in a 2 × 2 table.

The EID events used were sampled systematically from the list of 335 EID events identified by Jones et al. (*3*), starting from the most recent and going back until the required sample size was reached. The study required 160 events to have CIs that did not exceed 10% on each side of the point estimate of sensitivity and specificity if sensitivity was 90%, specificity was 55%, and 40% of events were truly of international public health concern. These values were chosen on the basis of the best available information (*9**,**10*). The IHR Annex 2 decision instrument was used to decide whether each EID event fulfilled the notification criteria. The decision was based on the information available in the references for each EID event provided in the original report by Jones et al. (*3*).

To emulate real-life conditions, the investigator used only information available at the time of event occurrence. Each criterion was answered by yes or no, and >2 positive answers classed the EID event as notifiable, according to WHO guidance. To establish the true outcome for every EID event, each expert had to give an opinion on 4 statements: the public health effect of the event was serious; the event was unusual or unexpected; the event spread internationally; and the event led to travel or trade restrictions. The 4 statements were derived from the IHR Annex 2 criteria, but were retrospective and ascertained the a posteriori outcome of each EID event. A Likert scale was used to score each statement with scores from 1 (strongly disagree) to 5 (strongly agree).

Experts based their decisions on their opinion and knowledge and on a supplied information sheet for each event. They were blinded to the notification outcome of each EID event and assessed each event independently. The opinion on each statement of each event for each expert was converted to a numerical score from −2 to +2 (Table 1), which was then summed to give an overall value for each statement and 4 values per EID event. For each statement, an overall positive score was considered a consensus agreement with the statement, and an overall negative score was considered a consensus disagreement with the statement. A null score was considered a failure to agree on that criterion. Events with >2 agreed statements were considered to be of international public health concern. Events with <1 agreed statement and >1 disagreed statement were considered to be of no international public health concern. Events for which there was 1 agreed statement and for which no agreement could be reached on 3 statements were not used in the study.

**Table 1:** Values allocated to each opinion for consensus building for emerging infectious diseases reported to WHO*

Opinion	Value allocated
Strongly disagree	−2
Somewhat disagree	−1
Neither agree nor disagree	0
Somewhat agree	+1
Strongly agree	+2

*WHO, World Health Organization.

Statistical analysis was performed by using Stata version 11 (StataCorp LP, College Station, TX, USA). A description was made of the distribution of events according to WHO region, type of pathogen, and type of event. We calculated the notification rate; the prevalence of EID events of international public health concern according to the expert panel; and the distribution of these events by type of pathogen, WHO region, and type of event. Sensitivity, specificity, PPV, and CIs of the decision instrument were then calculated. Concordance and its association with type of event, type of pathogen, and WHO region were calculated by using logistic regression. Concordance for each of the 4 criteria was also calculated. An intraclass correlation coefficient (*11*) was calculated for the combined score allocated by each expert (aggregated scores of all 4 criteria for each event, which provided a measure of overall concern; possible score of 20) to each EID event.

The appropriateness of the consensus-building method was tested by translating the judgment of each expert panel member into a binary scoring system, in which for each criteria, a score of 4 or 5 would translate to “I agree” and a score of 1, 2, or 3 would translate to “I disagree.” This process enabled identification of which EID events experts individually considered to be of public health concern. EID events with >2 criteria agreed with signifying international public health concern. Agreement levels between individual experts and the consensus were then calculated.

## Results

Of 204 identified EID events, 13 were not eligible because they did not fit the definition of an EID or were duplicates. Sixteen events were discarded because of insufficient information, leaving 175 (92%) of 191 eligible events to be analyzed. Their characteristics are summarized in Table 2. A total of 124 (70.9%) of 175 events fulfilled >2 of the 4 decision instrument criteria according to the notifying investigator and should have been reported to WHO, according to the Annex 2 decision instrument. No EID event was withdrawn from the study because of failure of the expert panel to agree. Of the 175 EID events assessed by the expert panel, 46 (26.3%) were deemed to be of international public health concern. Characteristics of these 46 events are shown in Table 3.

**Table 2:** Characteristics of 175 EID events reported to WHO*

Characteristic	No. (%)
Type of pathogen	
Bacterium	89 (50.9)
Virus	39 (22.3)
Rickettsia	12 (6.9)
Protozoan	20 (11.4)
Helminth	2 (1.1)
Fungus	12 (6.9)
Prion	1 (0.6)
WHO region	
AMRO	77 (44.0)
EURO	49 (28.0)
EMRO	7 (4.0)
SEARO	10 (5.7)
WPRO	24 (13.7)
AFRO	8 (4.6)
Type of event	
New pathogen	70 (40.0)
Increased incidence	44 (25.1)
Antimicrobial drug resistance	54 (30.9)
New clinical manifestation	7 (4.0)

*EID, emerging infectious disease; WHO, World Health Organization; AMRO, Americas; EURO, Europe; EMRO, Eastern Mediterranean; SEARO, Southeast Asia; WPRO, Western Pacific; AFRO, Africa.

**Table 3:** Characteristics of 46 EID events of international public health concern reported to WHO*

Characteristic	No. (%)
Type of pathogen	
Bacterium	21 (45.6)
Virus	18 (39.1)
Rickettsia	2 (4.3)
Protozoan	3 (6.5)
Other†	2 (4.3)
WHO region	
AMRO	17 (37.0)
EURO	12 (26.1)
EMRO	3 (6.5)
SEARO	2 (4.3)
WPRO	7(15.2)
AFRO	5 (10.9)
Type of event	
New pathogen	19 (41.3)
Increased incidence	18 (39.1)
Antimicrobial drug resistance	8 (17.4)
New clinical manifestation	1 (2.2)

*EID, emerging infectious disease; WHO, World Health Organization; AMRO, Americas; EURO, Europe; EMRO, Eastern Mediterranean; SEARO, Southeast Asia; WPRO, Western Pacific; AFRO, Africa. †Helminth, prion, or fungus.

Of 46 EID events of international public health concern, 44 would have been reported by using the Annex 2 decision instrument (sensitivity 95.6%; 95% CI 89.8%–100%). Of the 129 EID events that were not of international public health concern, 80 would still have been reported by using the Annex 2 decision instrument (specificity 38%, 95% CI 29.6%–46.3%; PPV 35.8%; 95% CI 27.1%–43.9%).

The overall concordance rate between notification decision and international public health concern was 53.1% (95% CI 45.7%–60.5%). The concordance rates for the 4 criteria of seriousness, unusualness, international spread, and travel and trade restrictions were 49.7% (95% CI 42.3%–57.1%), 58.3% (95% CI 51.0%–65.6%), 81.1% (95% CI 75.3%–86.9%) and 96% (95% CI 93.1–98.9), respectively. There was no strong evidence that the type of pathogen, type of event, or WHO region was associated with concordance (Table 4).

**Table 4:** Association between concordance and pathogen type, by WHO region and event type*

Characteristic	No. events	No. (%) concordant events	Crude OR (95% CI)	p value†
Type of pathogen				
Bacterium	89	27 (69.2)	1	
Virus	39	46(51.7)	2.10 (0.95–4.67)	0.07
Rickettsia	12	6 (50.0)	0.93 (0.28–3.12)	0.91
Protozoan	20	7 (35.0)	0.50 (0.18–1.38)	0.18
Other‡	15	7 (46.7)	0.82 (0.27–2.45)	0.72
WHO region				
AMRO	77	40 (51.9)	1	
EURO	49	23 (46.9)	0.82 (0.40–1.68)	0.58
EMRO	7	4 (57.14)	1.23 (0.26–5.88)	0.79
SEARO	10	5 (50.0)	0.92 (0.25–3.45)	0.91
WPRO	24	15 (62.0)	1.54 (0.60–3.94)	0.37
AFRO	8	6 (75.0)	2.78 (0.53–14.6)	0.23
Type of event				
New pathogen	70	31 (44.3)	1	
Increased incidence	44	27 (61.4)	2.00 (0.93–4.31)	0.08
Antimicrobial drug resistance	54	32 (59.3)	1.83 (0.89–3.75)	0.10
New clinical manifestation	7	3 (42.9)	0.94 (0.20–4.53)	0.94

*WHO, World Health Organization; OR, odds ratio; AMRO, Americas; EURO, Europe; EMRO, Eastern Mediterranean; SEARO, Southeast Asia; WPRO, Western Pacific; AFRO, Africa. †By Wald test. ‡Helminth, prion, or fungus.

The intraclass correlation coefficient for assessing the agreement level for overall public health concern for each event, by using an aggregated score of 20, was 0.68 (95% CI 0.60–0.74). After simplifying the scores to obtain a judgment for each EID event for each expert, the agreement levels for each panel member compared with those of the consensus were 76.5%, 84.6%, and 85.7%, respectively.

## Discussion

The IHR Annex 2 decision instrument has a high sensitivity (95.6%; 95% CI 89.8%–100%) but a low specificity (38%; 95% CI 29.6%–46.3%). These figures are consistent with previous anecdotal evidence (*9**,**10*). In this situation, trading specificity for high sensitivity is desirable because missing events of international public health concern would have serious consequences and would outweigh benefits of a lower volume of false-positive results. A low specificity is not a major concern as long as the volume of notification is low (*9*), and currently there is “little evidence that Annex 2 is being frequently or routinely used by State Parties in the assessment of events“ (*12*). A low specificity could become problematic if the volume of events reported through Annex 2 increased. The low specificity would result in an increase in false-positives results and increased costs associated with the notification process and determination of serious events.

The low specificity is reflected in a PPV of 35.8%. The calculated PPV could be underestimated for 2 main reasons. First, the prevalence of events identified as being of international public health concern might not reflect the prevalence of events truly reported to WHO. Second, in the current study, all EID events selected were submitted to the decision instrument, regardless of personal judgment. In real life conditions, events least likely to be of international public health concern would have been excluded even before being submitted to the decision instrument, which would increase the prevalence of events of international public health concern in events submitted to the decision instrument and consequently the PPV.

The specificity estimate was lower than that in 2 other evaluations (*9**,**10*) (38% vs. 50% and 55%, respectively). Although our estimate could be a more accurate reflection of the instrument specificity, it could also be an underestimate. Because instrument criteria are quite flexible and subject to interpretation, it is possible to reach a decision to report an event in which the likelihood of it becoming of international public health concern is small. In addition, courtesy bias, in which the assessor believes that that erring on the side of caution is more acceptable than not reporting that an event, could have occurred.

The current study strictly applied the criteria described in the Annex 2 guidance without using the context of the event or personal judgment. The decision instrument criteria are designed to take context and personal judgment into account to be adaptable to current and future unknown threats (*13*). Use of personal judgment rather than strictly applying the decision instrument criteria leads to a lower notification rate (*9*).

Two events of international public health concern were missed despite the high sensitivity of the instrument, which reflected challenges of predicting evolution of an event as it occurs and potential for human error. Although a sensitivity of 100% would be difficult to attain, maintaining the number of missed events at an absolute minimum should be a priority when the instrument is revised or evaluated.

Prediction of seriousness and unusualness of events were least accurate and showed concordance rates of 49.7% and 58.3%, respectively. This finding reflects the subjectivity and broad spectrum of the seriousness and unusualness criteria. Although these findings might lead to overreporting, criteria flexibility is also “a major strength that makes the IHR future-proof against new and unforeseeable threats” (*9*). The other 2 criteria of international spread and restriction to travel and trade have higher concordance rates of 81.1% and 96%, respectively. Should there be a need to increase the specificity of the instrument, the focus should be on tightening the first 2 criteria and one should be more specific about what makes an event serious or unusual. Training staff at NFPs could also increase the specificity of the instrument (by perfecting their use of the decision instrument) and its PPV (by prefiltering which events to submit to the decision instrument). Staff of NFPs have been trained in the past by using online tools and workshops (*10**,**14*), and both approaches could be used.

Sensitivity and specificity of the decision instrument did not depend on event type, pathogen type, or WHO region of occurrence because no strong evidence of an association between concordance and these factors was found. This finding suggests the Annex 2 decision tool is adequate for reporting antimicrobial drug resistance, although it was not designed with drug resistance in mind. There have been calls to use the decision instrument for antimicrobial drug resistance events (*15*).

Although EID events were systematically, rather than randomly, sampled from the EID list compiled by Jones et al. (*3*), the distribution of events by type of pathogen was not significantly different from the distribution of events in the complete list from which the study sample is extracted. The study sample and database from which it is extracted have a proportion of bacteria that is higher than other estimates of EID distribution (*16**,**17*). This finding can be explained by the fact that a large (43.8%) proportion of bacterial events are antimicrobial drug resistance events, which were not included as EIDs in many other studies. Jones et al. also reported a bias toward events occurring in industrialized countries, which reflect publication bias and better surveillance systems in these countries (*3*). However, these findings do not affect the internal validity of the study, and the fact that the current sample includes a wide variety of types of events can give confidence that the types of EID events truly reported to WHO are likely represented in the study sample.

The 16 events for which no information could be obtained did not statistically differ from the rest of the events, and the proportion of events without information was relatively low (8%), which made bias caused by information availability unlikely. The notifying investigator could not be blinded to EID events he or she was assessing, and it was possible to identify famous EID (such as emergence of Nipah virus) from the information, potentially introducing a bias toward reporting famous events. However, knowledge of these events is often the result of international concern, and they would have been reported regardless of these factors.

The intraclass correlation coefficient of 0.68 showed moderate-to-strong levels of agreement between expert panel members. The overall score given by each judge for each event was believed to be a good overall reflection of the role of the event. One limitation of this method was that the same score could be obtained with different opinions: e.g., if 1 expert strongly agreed that an event was serious but strongly disagreed that an event spread internationally, it would produce the same score as another expert strongly disagreeing with seriousness but strongly agreeing with international spread. However, when agreement levels were assessed for each criterion by calculating 4 intraclass correlation coefficients, there was no strong disagreement on any of the criteria, making that scenario unlikely.

The method showed agreement levels between experts and the consensus >75%. This agreement could have been improved by using a Delphi style approach (*18*), showing panel members their results compared with the mean of the whole panel and having a second round of evaluation.

This study took the approach of treating the IHR decision instrument a as a screening tool, thus enabling screening evaluation methods to be applied. One strong point of this study was the sample size: 175 real life events, a large enough sample to accurately estimate sensitivity and specificity with relatively narrow CIs. Furthermore, the fact that retrospective events were used enabled testing for predictive validity because in hindsight it was possible to evaluate the true international public health role of each event rather than just its potential for international public health concern. All panel members were internationally recognized as experts in the field. Therefore, their opinions were as reliable as can be obtained by using such a method. The fact they were blinded to whether each event would be reported and to each other’s opinions, and the objective method used to decide on consensus for each EID event further strengthens the method. Increasing the size of the panel may also have added rigor to the evaluation.

The definition of an EID was wider and more encompassing than most definitions used in the literature, particularly because it included antimicrobial drug resistance. Therefore, the validity of the decision instrument was tested by using a wide variety of type of events likely to represent a range of events NFPs staff would encounter in real life.

This study attempted to replicate real-life situations by means of a theoretical exercise. The amount of information available on each event was limited, and the WHO Annex 2 decision instrument criteria described in the guidance were rigidly applied. Furthermore, political or economic considerations that could not be replicated in a study are often taken in account when reporting an event (*19*). Therefore, the study implies a degree of simplification of real-life conditions.

The sample of events was limited to EIDs in which the Annex 2 decision instrument is used for a variety of events, including radiation and chemical incidents and outbreaks of well-established pathogens. Whether the results of this study can be extrapolated to such events is not clear.

Although as much care as possible was taken to make the expert panel method objective, it still relied to some extent on individual opinion, and expert panel judgment on each event could not claim to be the definitive and universal truth. This shortcoming is inherent to the method and has been noted in other studies of the IHR Annex 2 decision instrument that used expert panels (*9**,**10*). Every attempt was made to minimize subjectivity by giving clear written guidelines to each expert, blinding the experts to the notification outcome, preventing experts from discussing the events, and deriving agreement mathematically.

The IHR Annex 2 decision instrument is a sensitive tool for reporting EIDs of international public health concern. The instrument lacks specificity mainly because of broad, nonspecific criteria that can lead to overreporting. The PPV of the instrument is also relatively low. If one considers the nature of the instrument and potential consequences of WHO not being aware of an EID event of international public health concern, sensitivity should be prioritized over specificity. In the current situation in which the volume of notification remains low, the instrument is adequate. However, if the IHR Annex 2 decision instrument is to be used more systematically in reporting of and the volume of notification increases, there may be a need to increase the specificity and PPV of the instrument. This increase could be achieved by focusing particularly on setting more prescriptive seriousness and unusualness criteria to be more specific about what constitutes a serious or an unusual event, and by regular training of NFP staff online and through workshops to ensure that NFP staff report only relevant events, which would improve specificity without decreasing sensitivity and in turn increasing PPV. Also, focus should be placed on keeping the number of missed events to a minimum. However, instrument criteria must retain a certain level of interpretability so that the instrument can be adapted to a variety of unknown threats in the future, and not sacrifice sensitivity, which should remain the priority of the instrument. Finally, the approach taken in treating the IHR decision instrument as a screening tool and evaluating it as such has proved useful in understanding its value and limitations.
